# Serum cholesterol selectively regulates glucocorticoid sensitivity through activation of JNK

**DOI:** 10.1530/JOE-14-0456

**Published:** 2014-11

**Authors:** Nan Yang, Giorgio Caratti, Louise M Ince, Toryn M Poolman, Peter J Trebble, Cathy M Holt, David W Ray, Laura C Matthews

**Affiliations:** 1 Manchester Centre for Nuclear Hormone Research in Disease and Institute of Human Development, Faculty of Medical and Human Sciences, University of Manchester, AV Hill Building, Oxford Road, Manchester, M13 9PT, UK; 2 Institute of Cardiovascular Sciences, Faculty of Medical and Human Sciences, University of Manchester, CTF Building, Grafton Street, Manchester, M13 9PT, UK

**Keywords:** glucocorticoid receptor, inflammatory disease, cholesterol, transcription factors, signal transduction

## Abstract

Glucocorticoids (Gc) are potent anti-inflammatory agents with wide clinical application. We have previously shown that increased serum concentration significantly attenuates regulation of a simple Gc-responsive reporter. We now find that glucocorticoid receptor (GR) regulation of some endogenous transactivated but not transrepressed genes is impaired, suggesting template specificity. Serum did not directly affect GR expression, activity or trafficking, implicating GR crosstalk with other signalling pathways. Indeed, a JNK inhibitor completely abolished the serum effect. We identified the Gc modulating serum component as cholesterol. Cholesterol loading mimicked the serum effect, which was readily reversed by JNK inhibition. Chelation of serum cholesterol with methyl-β-cyclodextrin or inhibition of cellular cholesterol synthesis with simvastatin potentiated the Gc response. To explore the effect *in vivo* we used *ApoE* ^−/−^ mice, a model of hypercholesterolaemia. Consistent with our *in vitro* studies, we find no impact of elevated cholesterol on the expression of GR, or on the hypothalamic–pituitary–adrenal axis, measured by dexamethasone suppression test. Instead we find selective Gc resistance on some hepatic target genes in *ApoE* ^−/−^ mice. Therefore, we have discovered an unexpected role for cholesterol as a selective modulator of Gc action *in vivo*. Taken together these findings reveal a new environmental constraint on Gc action with relevance to both inflammation and cancer.

## Introduction

Glucocorticoids (Gc) are potent anti-inflammatory agents and are widely prescribed for the treatment of a range of inflammatory and immune diseases including rheumatoid arthritis and asthma (McMaster & Ray 2007, Barnes & Adcock 2009, De *et al*. 2011). In humans cortisol is the principal circulating Gc, but additional modulatory molecules are present including cortisone and the 5α-reduced cortisol metabolites (McMaster *et al*. 2008, Nixon *et al*. 2012).

Natural and synthetic Gc molecules modulate the activity of the near ubiquitously expressed glucocorticoid receptor (GR). The GR is a member of the nuclear hormone receptor superfamily and acts as a ligand-inducible transcription factor by interacting with chromatin to regulate gene transcription (Ito *et al*. 2006, Schacke *et al*. 2007, Sommer & Ray 2008). Selection of GR binding sites across the genome is dependent on cell-type specific chromatin structure and activity of comodulator proteins which regulate the accessibility of GR to target DNA (Herrera *et al*. 1989, Biddie *et al*. 2011, John *et al*. 2011).

Patients' responses to Gc treatment may vary, which is a major factor that limits the therapeutic utility of Gc. Many human disorders are affected by Gc signalling; therefore, there is great interest in defining mechanisms underlying how local tissue Gc sensitivity is being regulated (Yang *et al*. 2012). Amongst the best-characterised alterations in local Gc sensitivity is the acquired resistance that occurs at foci of inflammation, which is attributed to multiple pathways including cytokine action and activation of transcription factors including nuclear factor kappa B (NFκB) and activator protein 1 (AP1) (Yang *et al*. 2012).

While developing a sensitive Gc bioassay, we identified a striking and unexplained effect of serum which reduced the ability of GR to transactivate a simple reporter gene (Perogamvros *et al*. 2011, 2012). Serum contains a complex mixture of nutritional and signalling components, as well as classical hormones. The powerful effect of serum components on transcriptional responses have been known for a number of years, primarily through the activation of serum response factor (SRF) acting at AP1 elements (Treisman 1986, Shaw *et al*. 1989). More recently, a blood-borne signal with powerful activity to reset cellular circadian oscillators was discovered. The circulating factor in this case was thought to be a polypeptide, as yet unidentified, but one capable of acting through serum-response factor (Gerber *et al*. 2013).

Herein, we define the role of serum components on regulating Gc sensitivity. We identify cholesterol as a critical Gc regulatory serum component, and also that activation of JNKs and AP1 is essential for mediating the cholesterol effect. We further show that modulating cellular cholesterol metabolism using statins increases cellular sensitivity to Gc action. Given that membrane cholesterol release in response to cell necrosis is found both in cancers and at sites of inflammation, this provides a new mechanism to explain how a local environmental signal can selectively modulate the Gc response.

## Materials and methods

Dexamethasone (Dex), water soluble cholesterol and methyl-β-cyclodextrin were purchased from Sigma. The following antibodies were used: anti-GR (mouse clone 41, BD Biosciences, San Jose, CA, USA); anti-(s211)phospho-GR, anti-JNK and anti-phospho JNK, from Cell Signalling Technology (Beverly, MA, USA); anti-caveolin-1 from Santa Cruz; anti-α-tubulin from Sigma; anti-β-actin from Abcam (Cambridge, UK); and HRP-conjugated anti-mouse and anti-rabbit from GE Healthcare (Little Chalfont, UK). Fluorophore-conjugated (Alexa Fluor 546 and 488) anti-mouse and anti-rabbit antibodies were Invitrogen.

Human WT GRα expression vector and pMMTV-Luc have been described before (Ray *et al*. 1996). TAT3-Luc (Iniguez-Lluhi *et al*. 1997) was a kind gift from K Yamamoto (University of California San Francisco, CA, USA). The pSG5–steroid receptor coactivator 1 (SRC1) expression vector (Chen *et al*. 1999) was the kind gift of M Stallcup (University of Southern California, CA, USA). interleukin 6 (IL6)-Luc reporters were obtained from the BCCM plasmid collection (Zwijnaarde, Belgium). Expression vectors of MKK7 activated JNK and ERK (Tsuruta *et al*. 2004) were kind gifts from A Sharrocks (University of Manchester). pcDNA5 (Invitrogen) was used as an empty control. CMV-Renilla (Promega) was used to correct for transfection efficiency.

### Animals

*ApoE* ^−/−^ mice have been described previously (Plump *et al*. 1992). Transgenic and WT (C57BL/6) mice were routinely housed in 12 h light:12 h darkness (L:D) cycles with *ad libitum* access to food and water in a pathogen-free animal facility at the University of Manchester. All experiments were carried out in strict accordance with the Animals (Scientific Procedures) act 1986.

Serum samples were collected from age matched (12–18 weeks) female WT (C57BL/6) and *ApoE* ^−/−^ mice at morning (0700 h) and evening (1900 h) from the sequential tail vein bleeds. The following day (1500 h), animals were weighed and injected with either Dex (1 mg/kg i.p.) or vehicle control. Four hours later (to coincide with peak corticosterone levels), mice were killed (pentobarbital i.p.) and trunk blood was collected for the assessment of serum lipid and corticosterone levels. The livers were snap frozen for protein and RNA analysis.

### Cell culture

Human epithelial carcinoma (HeLa) cells were obtained from the European Collection of Cell Cultures (Salisbury, UK) and maintained in DMEM supplemented with GlutaMAX I and 10% fetal bovine serum (FBS, Invitrogen), 10% charcoal stripped FBS (CSS, Gibco), or lipoprotein deficient FBS (LDS, Sigma) in a humidified atmosphere of 5% CO_2_ at 37 °C.

### Reporter gene assay

1×10^6^ cells were transfected with 2 μg of firefly luciferase reporter and 0.5 μg of CMV-Renilla luciferase using FuGENE 6 (Roche Diagnostics). After 24 h, the cells were pooled and transferred to 24 well plates, then treated before lysis, and assay for dual luciferase activity according to the manufacturer's instructions (Promega). Some experiments also required cotransfection with an expression plasmid. For these studies, 1 μg expression plasmid (SRC1, ERK, or JNK) was included in the reporter gene transfection mix. Where appropriate, total transfected DNA was maintained by including an empty vector control, pcDNA5. To control for transfection efficiency, firefly luciferase counts were normalised with CMV-driven Renilla luciferase. Dose–response curves were generated using GraphPad Prism (La Jolla, CA, USA).

### Quantitative RT (RT-PCR)

Total RNA was prepared from HeLa cells using the RNeasy Mini Kit with on column DNase I digestion (Qiagen), and from mouse livers using SV Total RNA Isolation System (Promega). cDNA was synthesised using a High Capacity RNA-to-cDNA Kit and analysed using Power SYBR Green PCR Master Mix (Applied Biosystems). A group of Gc-target genes were selected from our previous microarray expression studies (Donn *et al*. 2007). Quantitative RT-PCR primer sequences are provided as supplementary information (Supplementary Tables 1 and 2, see section on supplementary data given at the end of this article). Expression levels were calculated using the comparative *C*_t_ method, normalising with the GAPDH or β-actin control, as indicated in the results.

### Immunoblot analysis

HeLa cells and murine liver tissue (in FastPrep-24 lysing matrix tubes; MP Biomedicals, Santa Ana, CA, USA) were lysed using radio-immunoprecipitation assay buffer (50 mM Tris–HCl, pH 7.4, 1% NP40, 0.25% Na-deoxycholate, 150 mM NaCl, and 1 mM EDTA) containing protease (Calbiochem, San Diego, CA, USA) and phosphatase inhibitors (Sigma–Aldrich Corp.). The proteins were separated by SDS gel electrophoresis and transferred to 0.2 μM nitrocellulose membranes (Bio-Rad Laboratories) overnight at 4 °C. The membranes were blocked for 6 h (0.15 M NaCl, 1% milk, and 0.1% Tween 20) and incubated with primary antibodies (diluted in blocking buffer) overnight. Following three washes (88 mM Tris, pH 7.8, 0.25% dried milk, and 0.1% Tween 20), membranes were incubated with a species-specific HRP-conjugated secondary antibody (in wash buffer) for 1 h at room temperature and washed a further three times, each for 10 min. Immunoreactive proteins were visualised using ECL Advance (GE Healthcare).

### Immunofluorescence

The cells were fixed with 4% paraformaldehyde for 20 min at room temperature and permeabilised (0.25% Triton X-100 in PBS) for 5 min at room temperature. The cells were blocked (3% goat serum and 0.1% Triton X-100 in PBS) for 30 min and then in primary antibody (diluted in blocking buffer) overnight at 4 °C. After three 5 min washes in PBS, cells were incubated in the secondary antibody for 2 h. Following three further 5 min washes, coverslips were mounted using Vectasheild hard-set mounting compound containing the nuclear DAPI stain (Vector Laboratories, Burlingame, MA, USA). The images were acquired on a DeltaVision RT (Applied Precision, Issauah, WA, USA) restoration microscope using a ×60/1.42 Plan Apo objective and the Sedat filter set (Chroma 89000; Chroma Technology Corp., Rockingham, VT, USA). The images were collected using a CoolSNAP HQ (Photometrics, Tuscon, AZ, USA) camera with a Z optical spacing of 0.5 μm. The images were deconvolved using Softworx Software (GE Healthcare, Issaquah, WA, USA) and maximum intensity projections of images processed using Image J (http://imagej.nih.gov/ij/; National Institute of Health, Bethesda, MD, USA).

### Live cell imaging

Transfection was performed by seeding 1×10^6^ HeLa cells (Fugene 6) with 5 μg hGR–GFP. After 24 h, the cells were transferred to glass bottomed 24 well plates. They were maintained at 37 °C and 5% CO_2_ for the duration of data collection. The images were acquired on a Nikon TE2000 PFS microscope using a 60×/1.40 Plan Apo objective and the Sedat filter set (Chroma 89000) and images collected using a Cascade II EMCCD camera (Photometrics). Raw time series images were processed using Image J.

### Fluorescent recovery after photobleaching

Transfection was performed by seeding 1×10^6^ HeLa cells (Fugene 6) with 5 μg hGR–GFP overnight then transferred to glass bottomed 24 well plates. They were maintained at 37 °C and 5% CO_2_ for the duration of data collection. The images were collected on a Leica TCS SP5 AOBS inverted confocal using a 63×/0.50 Plan Fluotar objective and 7× confocal zoom. The confocal settings were as follows, pinhole 1 airy unit, scan speed 1000 Hz unidirectional and format 1024×1024. The images were collected using the following detection mirror settings; FITC 494–530 nm using the 488 nm (13%). Raw time series images were processed using Image J.

### Proliferation assay

The levels of proliferation were measured using Cell Titer 96 AQueous One Solution Cell Proliferation Assay (MTS, Promega) as per instructions provided in the kit.

### Corticosterone measurements

Corticosterone in serum samples was measured using enzyme immunoassay (ENZO Life Sciences Ltd, Farmingdale, NY, USA) as per manufacturer's instructions. All serum samples were diluted 1:40 and assayed individually due to the low volumes obtained. A standard (five-point, 20 000 pg/ml max) curve comprising fivefold serial dilutions was generated alongside test samples.

### Statistical analysis

Data were expressed as average±s.e.m. (of at least three independent triplicate experiments) and compared using SPSS Software (version 16, SPSS, Inc.). For more than two groups, means were compared by one-way ANOVA followed by Bonferroni's *post hoc* test, and for comparison of two groups a Student's *t*-test for independent samples or Mann–Whitney was used. *P*<0.05 was considered statistically significant.

## Results

### Serum impairs Gc action through a post-receptor mechanism

**Figure 1:**
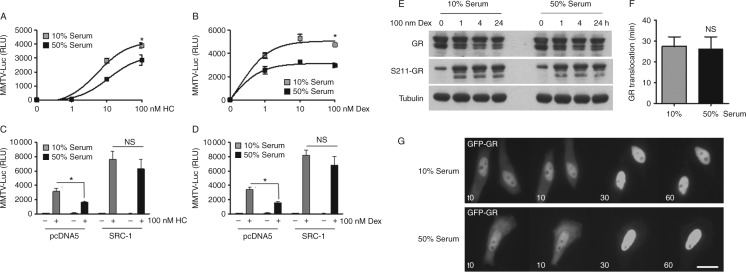
Serum factors induce Gc resistance. HeLa cells were transiently transfected with MMTV-Luc, Renilla-Luc and GR (A and B) together with pcDNA5 or SRC1 expression vectors (C and D). Following overnight culture in either 10 or 50% serum, cells were treated with hydrocortisone (HC) (A and C) or dexamethasone (Dex, 0–100 nM) (B and D) for 16 h before luciferase assay. Cells were cultured in either 10 or 50% serum, treated with 100 nM Dex for up to 24 h before immunoblot for GR and ^P^S211-GR (E). Tubulin was used as a loading control. Cells were transfected with GR–GFP and then cultured in either 10 or 50% serum overnight. Cells were treated with 100 nM Dex and imaged in real time. Images were acquired every 5 min. Average time for GR nuclear translocation in either 10 or 50% serum was calculated and presented in (F). Representative images are shown (G). Scale bar, 50 μM. Experiments were performed in triplicate and repeated three times. Graphs show mean±s.e.m. *P<0.05.

GR is a ligand-activated transcription factor. After nuclear translocation, ligand-bound GR dimerises and binds directly to DNA sequences to activate target gene expression. To study serum regulation of Gc action, synthetic reporter assays were performed using the GR-responsive reporter gene *MMTV*-Luc. Higher serum concentration (50%) impaired GR transactivation of MMTV-Luc in response to treatment with either Dex or hydrocortisone, demonstrable by a markedly decreased maximum response (reduced efficacy) (Fig. 1A and B). Overexpression of SRC1, which binds the GR to potentiate GR transactivation, rescued GR transactivation, suggesting a reversible model of impaired Gc action (Fig. 1C and D).

To determine whether the serum effect was a consequence of modulating GR expression or phosphorylation, immunoblot analysis was performed using global and phospho state-specific antibodies. In response to Dex treatment, steady state GR expression decreases, together with an increase in GR^S211^ phosphorylation, evident by 1 h (Fig. 1E). Culture in 50% serum did not affect either the expression or phosphorylation of the GR. Serum concentration also did not alter cell morphology, steady-state GR localisation or Dex-induced nuclear translocation (Supplementary Fig. 1, see section on supplementary data given at the end of this article). To investigate the rate of GR subcellular trafficking with better temporal resolution, live cell imaging was also completed. Serum had no effect on the rate of Dex-induced GR nuclear translocation (Fig. 1F and G). FRAP studies also identified no serum effects on GR intranuclear mobility either unliganded or after Dex treatment (Supplementary Fig. 2). Collectively, these studies suggest that the serum effect is not due to a direct regulation of GR expression, modification, or subcellular trafficking.

### Serum selectively and reversibly impairs GR transactivation of endogenous gene expression

**Figure 2:**
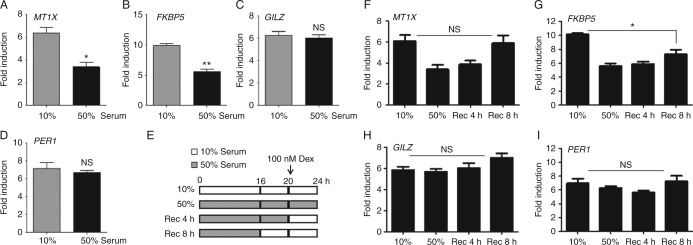
Serum impairs GR transactivation in a reversible manner. HeLa cells were cultured in either 10 or 50% serum overnight, then treated with 100 nM Dex for 4 h before RNA purification. Transcripts of four well-characterised GR transactivated endogenous genes FK506 binding protein 5 (*FKBP5*), metallothionein 1X (*MT1X*), glucocorticoid-induced leucine zipper (*GILZ*), and period circadian protein homolog 1 (*PER1*) were measured by qRT-PCR (all normalised to GAPDH) (A, B, C and D). Cells were either continually cultured in either 10 or 50% serum, or cultured in 50% serum then returned to 10% serum as shown in (E) before treatment with 100 nM Dex for 4 h and transcript analysis for *MT1X* (F), *FKBP5* (G), *GILZ* (H), and *PER1* (I). Experiments were performed in triplicate and repeated three times. Graphs show mean±s.e.m. *P<0.01 and **P<0.001. NS, not significant.

QRT-PCR was used to explore serum regulation of four well-characterised GR transactivated genes, metallothionein 1X (*MT1X*), FK506 binding protein 5 (*FKBP5*), Gc-induced leucine zipper (*GILZ, TSC22D3*), and period circadian protein homolog 1 (*PER1*). Following 4 h Dex treatment, all four genes were robustly induced: 50% serum impaired transactivation of both *FKBP5* and *MT1X* (Fig. 2A and B) whereas expression of *GILZ* and *PER1* was comparable in 10% and 50% serum (Fig. 2C and D). There were no serum effects on baseline gene expression or cell proliferation (Supplementary Fig. 3, see section on supplementary data given at the end of this article), suggesting that serum selectively impairs Gc-induced GR activity.

Further qRT-PCR assays were completed to investigate whether the impairment of GR transactivation was recovered following return to culture in 10% serum (Fig. 2E). Impairment of *MT1X* transactivation was rapidly reversed (Fig. 2F), whereas *FKBP5* regulation was not recovered even after 8 h (Fig. 2G). This suggests the existence of different mechanisms regulating variation in Gc response, acting in a template-dependent manner.

**Figure 3:**
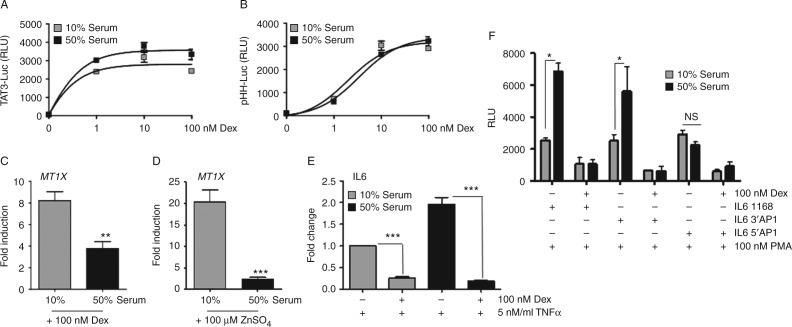
Serum increases GR transrepression of IL6 through AP1 elements. HeLa cells were transiently transfected with TAT3-Luc (A) or pHH-Luc (B), together with Renilla-Luc and GR. 24 h later, cells were transferred to culture in either 10 or 50% serum then treated with Dex for 16 h before luciferase assay. HeLa cells were cultured in either 10 or 50% serum overnight, and then treated with either 100 nM Dex (C) or 100 μM ZnSO_4_ (D) for 4 h before RNA purification and *MT1X* transcript measured by qRT-PCR (normalised to GAPDH). Cells were cultured in 10 or 50% serum overnight then treated with 5 ng/ml TNF for 1 h and Dex for a further 4 h. RNA was extracted and IL6 transcript measured by qRT-PCR (normalised to GAPDH) (E). HeLa cells were transiently transfected with WT or AP1 deletant IL6-Luc plasmids then cultured in 10 or 50% serum. Cells were treated with 100 nM PMA and 100 nM Dex for 16 h and then lysed for luciferase assay (F). Graphs show mean±s.e.m. Experiments were carried out in triplicate and repeated three times. *P<0.05, **P<0.01, and ***P<0.001. NS, not significant.

To define further response element specificity of serum action, two simple Gc response element (GRE)-reporter genes were examined. Serum had no effect on either the pTAT3-Luc, which contains tandem repeats of a simple, consensus GRE (Fig. 3A) or pHH-Luc, which is a truncated MMTV promoter containing just the GR binding elements (Fig. 3B). These findings suggest that target elements may be less important than the flanking, contextual DNA around the GR binding site for mediating the serum effect.

As expression of *MT1X* is transactivated by Zn ions as well as Gc, the serum effect of Zn transactivation on *MT1X* was also determined. The cells cultured in 10% serum had an eightfold induction of *MT1X* after treatment with Dex, and a 17-fold induction in response to ZnSO_4_ treatment. However, both of these robust transcriptional effects were dramatically decreased following culture with 50% serum, with only three- and twofold induction of *MT1X* respectively (Fig. 3C and D). This further suggests that the response element contextual DNA is more important for the serum effect than the GR, or the GR binding site itself, a conclusion strengthened by observing that GR repression of IL6 (Fig. 3E), MIF-Luc and IL8 (Supplementary Fig. 4, see section on supplementary data given at the end of this article) were in fact augmented by culture in 50% serum. To investigate Gc repression of the *IL6* gene in more depth, additional reporter assays were completed using a series of human IL6 luciferase constructs. Consistent with regulation of the endogenous gene, Gc repression of IL6-Luc was increased in 50% serum. Interestingly, this augmented Gc repression was lost when the 5′AP1-binding site was deleted (Fig. 3F), implicating AP1 action as part of the mechanism.

### Serum regulates Gc action through MKK7-activated JNK

**Figure 4:**
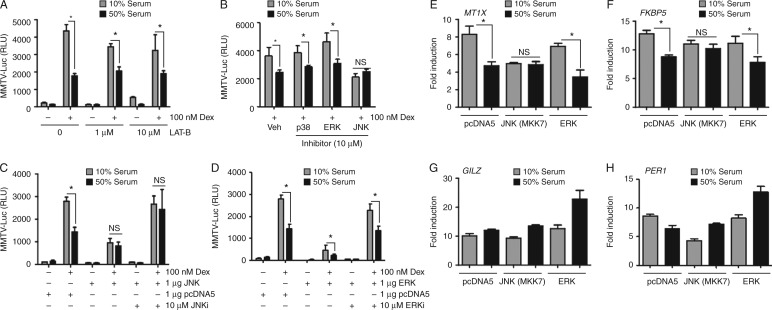
Serum impairs Gc action through activation of JNK. HeLa cells were transiently transfected with MMTV-Luc and GR overnight and then cultured in 10 or 50% serum. The cells were treated with Latrunculin B (A) or MAP kinase inhibitors (B) (ERK PD98059, p38 SB203580, and JNK SP600125) together with 100 nM Dex for 6 h before luciferase assay. The cells were transiently transfected with MMTV-Luc and GR, together with JNK (C) or ERK (D) expression vectors (or pcDNA5 as a control) overnight. The cells were transferred to 10 or 50% serum, treated with 100 nM Dex and 10 μM JNK inhibitor (SP600125) or ERK inhibitor (PD98059) for 6 h and then assayed for luciferase activity. HeLa cells were transfected with JNK or ERK expression vectors (or pcDNA5 as a control), cultured in 10 or 50% serum, and then treated with 100 nM Dex for 4 h before RNA extraction. Expression of *MT1X* (E), *FKBP5* (F), *GILZ* (G), and *PER1* (H) transcripts were measured by qRT-PCR (all normalised to GAPDH). The experiments were carried out in triplicate and repeated three times. Graphs show mean±s.e.m. *P<0.05. NS, not significant.

To delineate the signalling mechanism responsible for modulating the Gc response, a series of reporter gene studies were performed in conjunction with small molecule inhibitors. First, Gc-activated expression of *MMTV* reporter gene was measured following treatment with Latrunculin-B, a drug that sequesters G-actin and inhibits activation of SRF (Gineitis & Treisman 2001, Gerber *et al*. 2013). There was no effect on the serum induction of Gc resistance, suggesting a mechanism independent of SRF activation (Fig. 4A). To explore the role of MAP kinases, which activate AP1, in mediating repression of GR transactivation, cells were treated with three MAPK inhibitors. Inhibition of JNK reversed the inhibitory effect serum, whereas inhibition of either ERKs or p38 had no impact (Fig. 4B). To further specify the role of JNK, cells were transfected with either JNK or ERK expression vectors. Hyperactivation of JNK signalling attenuated GR transactivation and mimicked the serum effect, such that culture in 50% serum had no additional effect. Sensitivity to Gc could be restored by co-treatment with JNK inhibitor (Fig. 4C). Although overexpression of ERK alone also impaired Gc action, an inhibitory effect of serum was still observed suggesting coincident activation of parallel unrelated pathways (Fig. 4D).

To define the role of JNK on endogenous gene regulation, again cells were transfected with activated JNK and then analysed by qRT-PCR. As expected both *MT1X* and *FKBP5* were affected by serum concentration, in a JNK-dependent manner (Fig. 4E and F) and, induction of *PER1* or *GILZ* expression by Dex was not affected by serum or activation of JNK (Fig. 4G and H).

### Lipid components within serum impair Gc action

**Figure 5:**
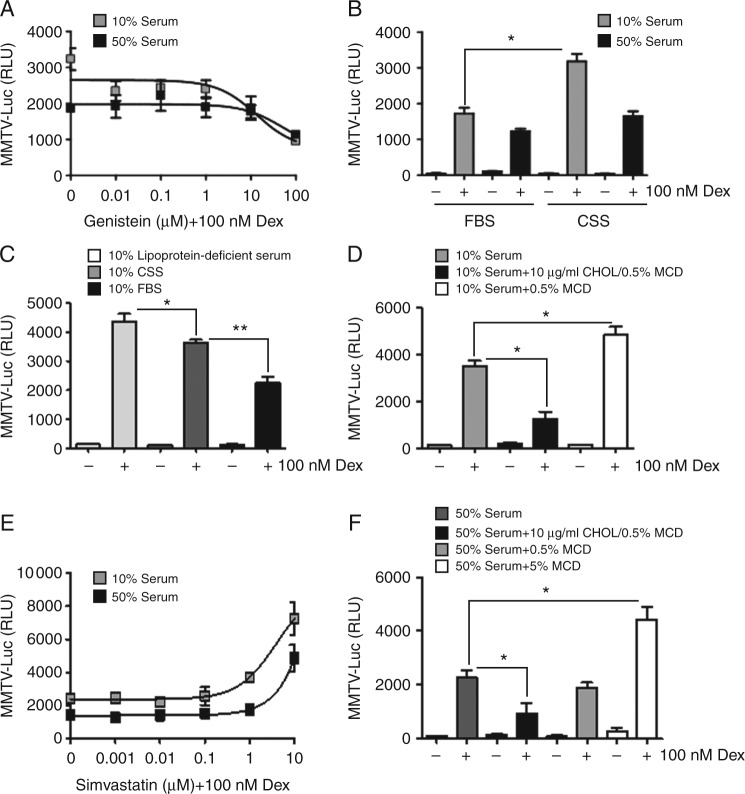
Modulation of cellular cholesterol regulates Gc effects. HeLa cells were transiently transfected with MMTV-Luc reporter gene and GR then transferred to 10 or 50% serum overnight. Cells were treated with Genistein (as indicated) for 4 h, and then treated with 100 nM Dex for a further 4 h before lysis and luciferase assay (A). Cells were also cultured in either normal serum, charcoal stripped serum or lipoprotein-deficient serum (B and C), and then treated with 100 nM Dex for 16 h before luciferase assay. Cells were cultured in 10% serum with 10 μg/ml cholesterol (CHOL) complexed with 0.5% methyl-β-cyclodextrin (MCD) or with 0.5% MCD alone (D). Cells transfected with MMTV-Luc and GR, cultured in 10 or 50% serum were treated with Simvastatin (as indicated) together with 100 nM Dex for 16 h before luciferase assay (E). Cells were cultured in 50% serum, together with cholesterol complexed with 0.5% MCD, 0.5%, or 5% MCD alone, then treated with 100 nM Dex for 16 h before luciferase assay (F). Experiments were carried out in triplicate and repeated three times. Graphs show mean±s.e.m. *P<0.05 and **P<0.01.

We show that culture in 50% serum selectively impairs GR transactivation through modulation of JNK, and sought to identify the component in serum responsible for mediating the effect. Serum contains many growth factors that signal through tyrosine kinase pathways. Treatment with genistein, a general inhibitor of tyrosine kinases, had no effect on serum induction of Gc resistance (Fig. 5A). In addition neither heat treatment, nor trypsin digestion of serum impaired the inhibitory effect of serum on GR action (Supplementary Fig. 5, see section on supplementary data given at the end of this article). In search of serum metabolites capable of regulating Gc function, activated charcoal was used to ‘strip’ small molecules. Culture in 10% charcoal stripped (delipidated) serum-permitted enhanced GR transactivation at 10%, and had no effect at 50% compared with full serum (Fig. 5B).

To further analyse the possible role of serum lipids in the regulation of Gc action, lipoprotein-deficient serum (containing <5% of normal lipoprotein) was also used. Culture in the reduced lipoprotein serum further increased the Gc response, implicating serum lipids in negatively regulating Gc responses (Fig. 5C). To pursue a possible role for cholesterol, cells were loaded with exogenous cholesterol (25 μmol/l, in a water-soluble complex with 0.5% methyl-β-cyclodextrin), or with 0.5% methyl-β-cyclodextrin alone (Fig. 5D). Cholesterol loading decreased GR transactivation, and treatment with methyl-β-cyclodextrin increased Gc action. Treatment with Simvastatin, which inhibits cholesterol synthesis, also potentiated Gc transactivation in either 10 or 50% serum (Fig. 5E). Importantly, methyl-β-cyclodextrin increased GR action in a concentration-dependent manner, where a higher concentration of methyl-β-cyclodextrin (5%) was necessary to quench the higher concentration of cholesterol present in 50% serum (Fig. 5F). The same high concentration of methyl-β-cyclodextrin was toxic to cells cultured in 10% serum overnight, presumably a consequence of quenching available serum cholesterol and also extracting cholesterol from cell membranes and inducing apoptosis. Importantly, we found only modest effects of Simvastatin, cholesterol or methyl-β-cyclodextrin treatment on cell proliferation or viability, excluding a possible cell cycle-dependent effect (Supplementary Fig. 6A, see section on supplementary data given at the end of this article). We also demonstrate that treatment with 5% methyl-β-cyclodextrin, which is able to bind Dex, does not alter the onset of GR translocation thereby excluding any direct effect of methyl-β-cyclodextrin on uptake of Dex by the cells or on the timing of GR activation (Supplementary Fig. 6B).

### Cholesterol selectively impairs GR transactivation through activation of JNK

**Figure 6:**
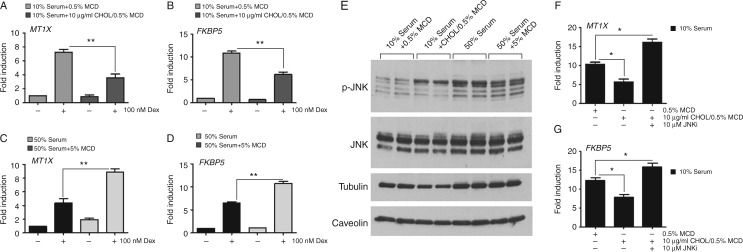
Cholesterol regulates GR transactivation via activation of JNK. HeLa cells were cultured in 10% serum or 10% serum supplemented with 10 μg/ml cholesterol overnight (A and B), or with 50% serum or 50% serum supplemented with 5% MCD (C and D). Cells were harvested after 4 h treatment with either 100 nM Dex or DMSO and analysed by qRT-PCR for expression of *MT1X* and *FKBP5* (normalised to GAPDH). HeLa cells cultured in 10% serum supplemented with 10 μg/ml cholesterol (CHOL) or 50% serum supplemented with 5% MCD overnight, and then protein lysates analysed by immunoblot for JNK and phosphorylated JNK (E). Tubulin and caveolin expression was used as loading controls. Cells were cultured in 10% serum supplemented with 10 μg/ml cholesterol, together with 10 μM JNK inhibitor (SP600125) for 6 h. Following treatment with 100 nM Dex for an additional 4 h, expression of GR transactivation of either *MT1X* (F) or *FKBP5* (G) was measured by qRT-PCR (normalised to GAPDH). Graphs show average±s.e.m. Experiments were carried out in triplicate and repeated three times. *P<0.05 and **P<0.001.

To investigate the cholesterol effect on GR transactivation of endogenous genes, the previous group of GR-transactivated genes *MT1X*, *FKBP5*, *GILZ*, and *PER1* were measured by qRT-PCR. After 4 h treatment with Dex, incubation with exogenous cholesterol inhibited induction of *MT1X* and *FKBP5* (Fig. 6A and B), but had no effect on *GILZ* or *PER1* in cells cultured in 10% serum (Supplementary Fig. 7A, see section on supplementary data given at the end of this article). Treatment with 5% methyl-β-cyclodextrin abolished the impact of 50% serum on GR transactivation of *MT1X* and *FKBP5* (Fig. 6C and D), again with no effect on *GILZ* or *PER1* (Supplementary Fig. 7B). There was no cholesterol effect on the baseline expression of these endogenous genes (Supplementary Fig. 7C). The liver X receptor (LXR, NR1H3) antagonist GSK1440233A did not reverse the effect of cholesterol, excluding the action of oxidised cholesterol through the *LXR*.

To determine whether the mechanism of cholesterol action was the same as that seen for culture in high-serum alone, through activation of JNK, immunoblot analysis was performed. Indeed, both high serum and cholesterol loading increased phosphorylation of JNK, a marker of increased activity. The serum-induced activation of JNK was attenuated by the addition of 5% methyl-β-cyclodextrin (Fig. 6E). The impaired GR transactivation of both *MT1X* and *FKBP5* after treatment with cholesterol was rescued by cotreatment with a JNK inhibitor (Fig. 6F and G), which confirms the requirement of JNK signalling in mediating the regulatory effect of cholesterol.

### Cholesterol selectively impairs GR transactivation *in vivo*

**Figure 7:**
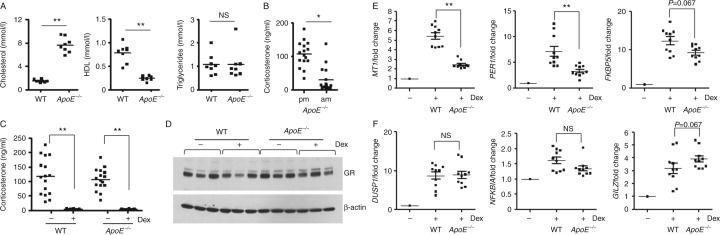
Cholesterol selectively regulates GR transactivation *in vivo*. Serum samples from WT and *ApoE* ^−/−^ mice (eight animals per group) were analysed for levels of cholesterol, HDL and triglycerides (A). Morning (0700 h) and evening (1900 h) serum samples from *ApoE* ^−/−^ mice (16 animals per group) were assayed for corticosterone (B). WT and *ApoE* ^−/−^ mice were subjected to a dexamethasone (Dex) suppression test. Mice (eight animals per group) were given i.p. injection of Dex (1 mg/kg) and then serum collected 4 h later (1900 h). Serum samples were assayed for corticosterone (C). WT and *ApoE* ^−/−^ mice (five animals per group) were given i.p. injection of Dex (1 mg/kg) and then livers collected 4 h later. Livers were analysed for GR expression by immunoblot (D). Blots show samples from three different animals. Livers were also analysed for induction of GR target genes *MT1*, *PER1*, and *FKBP5* (E), *DUSP1*, *NFKBIA*, and *GILZ* (F) by qRT-PCR (all normalised to GAPDH). Graphs show mean±s.e.m. *P<0.05 and **P<0.01. NS, not significant.

To investigate the effect of high cholesterol on GR transactivation *in vivo*, we studied Gc sensitivity in *ApoE* ^−/−^ mice (Plump *et al*. 1992). These mice have high serum cholesterol (Fig. 7A) but have rhythmic corticosterone levels within the normal range (Fig. 7B). We performed a Dex suppression test to measure ‘systemic’ Gc sensitivity and found no difference in response between WT and *ApoE* ^−/−^ mice (Fig. 7C). Analysis of protein from the livers of WT and *ApoE* ^−/−^ mice identifies no differences in GR expression (Fig. 7D). We next examined the impact of high serum cholesterol on GR transactivation *in vivo*. Consistent with our findings in cell lines, we find target-specific resistance to Dex treatment in *ApoE* ^−/−^ mice. Induction of MT1 and Per1 following i.p. Dex (1 mg/kg, 4 h) was attenuated in *ApoE* ^−/−^ mice, and although induction of *FKBP5* was reduced, this did not reach statistical significance (Fig. 7E). We found no effect of cholesterol on Dex-dependent induction of dual specificity phosphatase 1 (*DUSP1*), NFκB inhibitor alpha (*NFKBIA*) or *GILZ* (Fig. 7F). High circulating cholesterol therefore selectively modulates GR function *in vivo*.

## Discussion

Optimising Gc treatment is a challenge, a consequence of wide variation in host sensitivity, localised resistance at sites of inflammation, and undesired effects attributed to long-term use. The GR is ubiquitously expressed, and therefore obtaining selectivity of action is difficult. Developing selective GR modulators to improve the therapeutic index of Gc represents an essential step to improve existing Gc therapies (Beck *et al*. 2009). However, as the precise mechanism by which Gcs mediate their range of anti-inflammatory effects remains unclear, attempts to identify new molecules with selective action has proven difficult (Sommer & Ray 2008). Given the widespread therapeutic application of synthetic Gc in healthcare, understanding how target cell sensitivity to Gc is regulated is of major interest. While developing a Gc bioassay we discovered a potent effect of human serum on target cell response to Gc (Perogamvros *et al*. 2011). Serum is a complex mixture of nutrients and signalling molecules (hormones, cytokines, and growth factors) and new discoveries are still being made identifying novel physiological functions for serum in signalling to target tissues, such as providing time-of-day information (Gerber *et al*. 2013).

Herein, we show that serum impairs Gc action in a template-specific manner, where high serum impairs GR transactivation on some targets, but paradoxically augments GR transrespression of others. Our studies did not reveal any differences in GR expression, phosphorylation, trafficking, or intranuclear mobility either at steady state or following addition of Gc. These results suggest that the gene-selective mechanism of action of serum results from parallel activation of other signalling cascades and convergence on target genes based on the presence of transcription factor binding sites for GR, and other factors lying in such a way as to permit cross-talk. We investigated several endogenous GR-transactivated genes. Gc-induced expression of *MT1X* and *FKBP5* was attenuated by serum, while *GILZ* and *PER1* remains unaffected. Interestingly, sequence analysis of the *MT1X* gene sequence showed both AP1 and GRE-binding sites in close proximity (Viarengo *et al*. 2000, Velazquez & Gariglio 2002). In response to heat shock, which induces activation of AP1, there was also reduced Gc transactivation of *MT1X* (Andrews *et al*. 1987). The transcription factor AP1 is dimeric, consisting of JUN and FOS family members in a variety of homodimeric and heterodimeric forms. Studies have previously defined a mutually antagonistic mode of interaction (Lucibello *et al*. 1990, Schule *et al*. 1990), but more recently, AP1 has emerged as a necessary pioneer factor for GR access to target sites in chromatin, suggesting a far more complex mechanism of interaction than previously thought (Biddie *et al*. 2011, Uhlenhaut *et al*. 2013).

AP1 is activated downstream of MAP kinases, and using JNK inhibitors and constitutive JNK expression studies we were able to show that the serum effect required activation of JNK, and, indeed, serum shock induced JNK activation. The action of JNK on GR activity and subcellular shuttling has been previously reported in other contexts (Rogatsky *et al*. 1998, Loke *et al*. 2006).

Having identified a serum effect activating JNK/AP1 as the mechanism acting on GR signalling, we started by considering serum components likely capable of activating JNK. In contrast to other recent studies that identified a protein factor as the modulatory serum component (Gerber *et al*. 2013), we were able to exclude a significant effect mediated by serum protein components, using heat-inactivation, trypsin digestion, and growth factor signalling tyrosine kinase inhibition approaches. Instead, we did discover a key role for serum lipid components. We show that the response to Gc was augmented in cells cultured in charcoal dextran-treated (delipidated) serum, and lipoprotein-deficient serum increased Gc action to an even greater extent. Adding cholesterol alone was sufficient to modify the Gc response, which is consistent with an early report that found high cholesterol blocked the antiproliferative actions of Gc in leukaemia cells (Madden *et al*. 1986). We also used the cholesterol-binding drug methyl-β-cyclodextrin to sequester serum cholesterol (Brown & London 2000, Simons & Toomre 2000), which not only increased Gc transactivation in 10% serum culture, but also rescued the impairment of Gc action by high serum. We found that loading with exogenous cholesterol alone was sufficient to activate JNK, and that loss of GR transactivation with cholesterol was rescued by cotreatment with a JNK inhibitor. Treatment with Gc is reported to inhibit cholesterol synthesis, and so this may provide an indirect mechanism to modify JNK signalling, and also modify cellular responses to subsequent Gc treatments (Johnston *et al*. 1980).

In humans, circulating cholesterol (∼100–200 mg/dl or 2.5–5 mmol/l) is typically higher than that present in FBS (∼30–50 mg/dl or 0.8–1.3 mmol/l) and there are also differences in lipoprotein content between species. To assess the role of cholesterol rather than lipoproteins, we delivered cholesterol directly to target cells in complex with methyl-β-cyclodextrin and also regulated local cholesterol production using statin treatment. These studies reveal a specific effect of cellular cholesterol content on Gc sensitivity. Given the widespread application of cholesterol regulating drugs, and their reported impact on parameters of inflammation (Krane & Wanner 2011, Rodriguez *et al*. 2012), this is likely a clinically relevant interaction.

To explore the effects of high cholesterol on Gc sensitivity *in vivo*, we analysed GR function in *ApoE* null, hypercholesterolaemic mice. Although globally sensitive to Gc treatment, we again find target-selective resistance to Dex in livers from *ApoE* ^−/−^ mice, although the specific genes affected were slightly different between human and murine models. Our *in vitro* and *in vivo* data provide strong evidence for a novel and important regulatory role for cholesterol in the regulation of Gc responses *in vivo*. Gc are key components of the stress response, and are necessary for regulation of immune and metabolic function. Our findings therefore have important implications for patients with dyslipidaemia, treated with statins, or indeed at foci of inflammation, or injury in healthy individuals where cholesterol is found at high concentration.

## Supplementary data

This is linked to the online version of the paper at http://dx.doi.org/10.1530/JOE-14-0456.

## Author contribution statement

N Y, C M H, D W R, and L C M designed the research; N Y, G C, L M I, P J T, T M P, and L C M performed the research and analysed the data; N Y, G C, L M I, D W R, and L C M wrote the paper.

## Supplementary Material

Supplementary Data
