# Supplementary material for: Serum cholesterol selectively regulates glucocorticoid sensitivity through activation of JNK
Source: J Endocrinol. 2014 Aug 26;223(2):155–66. doi: 10.1530/JOE-14-0456 (PMC4191185; doi:10.1530/JOE-14-0456)
Supplement: Supplementary Data [file supp_223_2_155__index.html]

Serum cholesterol selectively regulates glucocorticoid sensitivity through activation of JNK — Cholesterol regulates glucocorticoid sensitivity — Supplementary Data 

# Serum cholesterol selectively regulates glucocorticoid sensitivity through activation of JNK

## Supplementary Data

**Files in this Data Supplement:**

- Supplementary Figure 1 - **Serum does not alter GR translocation**. HeLa cells were seeded onto coverslips, cultured in either 10% or 50% serum overnight (A) then fixed and labeled with TRITC phalloidin (red) and nuclei counterstained with DAPI (blue). Two fields are shown. HeLa cells were also, cultured in either 10% or 50% serum overnight then treated with 100nM Dex for 0, 10, 30 or 60 min (B). Cells were then fixed and probed using specific antibodies for GR (green) and nuclei counterstained with DAPI (blue). Scale bar, 100μM. (PDF 66 KB)
- Supplementary Figure 2 - **Serum does not affect GR intranuclear mobility**. HeLa cells were transfected with GR-GFP overnight, transferred to culture in 10% or 50% serum, then treated with 100nM Dex or DMSO (Veh) for 4 hours before FRAP analysis. The fluorescent intensity of regions of interest were measured prior to photobleaching and each second thereafter. Graphs depict the mean percentage of total fluorescent intensity prebleach (t0). (PDF 32 KB)
- Supplementary Figure 3 - **Serum does not affect baseline expression of GR transactivated genes**. HeLa cells were cultured in either 10% or 50% serum overnight before RNA purification and expression of GILZ, PER1, FKBP5 and MT1X (normalized to GAPDH) measured by qRT-PCR (A). The same samples were analysed by qRT-PCR for GAPDH (normalized to βactin, B). HeLa cells were cultured in either 10% or 50% serum overnight and cell proliferation analysed by MTS assay (C), where graph depicts the percentage of proliferating cells compared to culture in 10% serum. Graphs show average ± SEM. Experiments were performed in triplicate and repeated at least two times. NS, not significant. (PDF 41 KB)
- Supplementary Figure 4 - **Serum enhances GR transrepression**. HeLa cells were transiently transfected with MIFG-Luc, Renilla-Luc and GR, cultured in 10% or 50% serum, and then treated with 0-100nM Dex for 16 hours before luciferase assay (A). HeLa cells were cultured in 10% or 50% serum supplemented with 5ng/ml TNF overnight. Cells were treated with 100nM Dex for 4 hours prior to qRT-PCR assay for IL-8 transcript (B). Graphs show mean ± SEM. Experiments were performed in triplicate. \* P<0.05 \*\*P<0.001. (PDF 30 KB)
- Supplementary Figure 5 - **Serum effects are not mediated by proteins**. HeLa cells were transiently transfected with MMTV-Luc and GR then cultured in serum (normal or charcoal dextran stripped) that had been subject to prior heat treatment for 1 hour (at the temperatures indicated) and diluted to either 10% or 50% with cell culture media. Cells were treated with Dex for 16 hours before luciferase assay (A). Alternatively, serum was pretreated with trypsin followed by trypsin inhibitor or with trypsin inhibitor followed by trypsin for 2 hours. Cells were treated with digested serum diluted to either 10% or 50% with cell culture media, then 100 nM Dex for 16 hours (B). Graphs show mean ± SEM. Experiments were performed in triplicate and repeated three times. (PDF 36 KB)
- Supplementary Figure 6 - **Modulation of Cholesterol has no effect on GR activation**. HeLa cells were cultured in either 10% serum supplemented with vehicle or Simvastatin, carrier or cholesterol, 50% serum or 50% serum supplemented with 5% MCD overnight and cell proliferation analysed by MTS assay (A). Graphs depict the percentage of proliferating cells compared to vehicle/carrier treated control. Graphs show mean ± SEM. Experiments were performed in triplicate and repeated two times. \*\*P<0.001, NS: Not significant. Cells were also pretreated with 5% MCD (1hr), then treated with 100nM Dex for 0, 10 or 30 min (B). Cells were then fixed and probed using specific antibodies for GR (green) and nuclei counterstained with DAPI (blue). Scale bar, 100μM. (PDF 56 KB)
- Supplementary Figure 7 - **Cholesterol has no effect on GR transactivation of GILZ or PER1**. HeLa cells were cultured in 10% serum alone or 10% serum supplemented with 10 μg/ml cholesterol overnight (A), 50% serum or 50% serum supplemented with 5% MCD (B). After 4 hours treatment with either 100 nM Dex or DMSO cells were harvested and analyzed by qRT-PCR for expression of GILZ and PER1 (normalized to GAPDH). All treatment groups were also analysed for expression of GAPDH (normalized to βactin, C). Graphs show mean ± SEM. Experiments were performed in triplicate and repeated three times. NS: Not significant. (PDF 49 KB)
- Supplementary Table 1 - (PDF 75 KB)
- Supplementary Table 2 - (PDF 75 KB)
