# Supplementary figures and images for: Serum cholesterol selectively regulates glucocorticoid sensitivity through activation of JNK
Source: J Endocrinol. 2014 Aug 26;223(2):155–66. doi: 10.1530/JOE-14-0456 (PMC4191185; doi:10.1530/JOE-14-0456)

**A**

10% SERUM

50% SERUM

FIELD 1

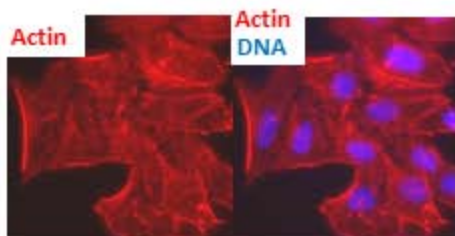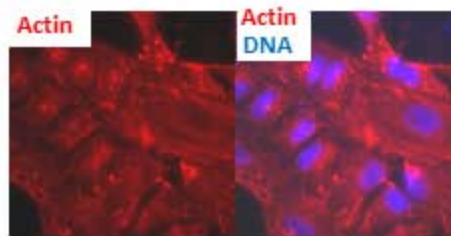

FIELD 2

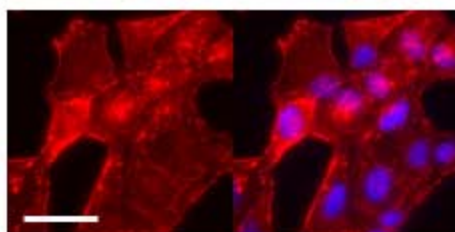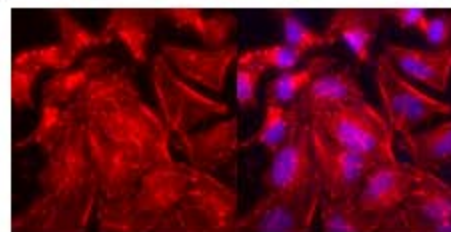**B**

10% SERUM

50% SERUM

CONTROL

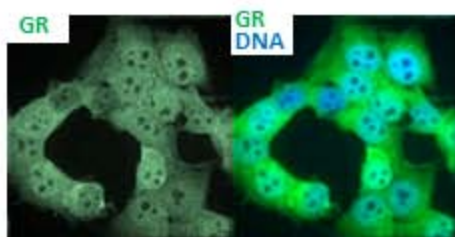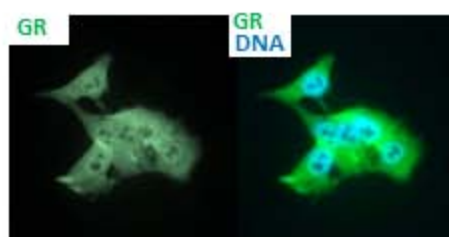

10 MINUTES

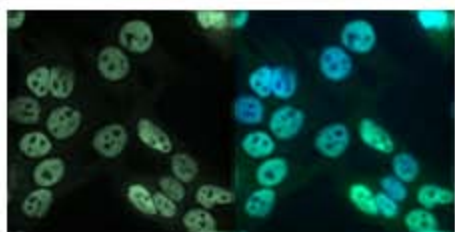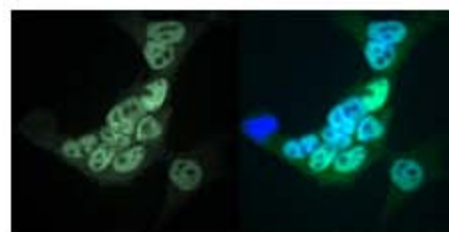

30 MINUTES

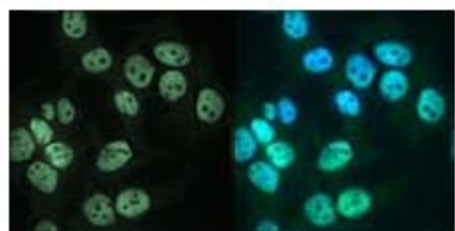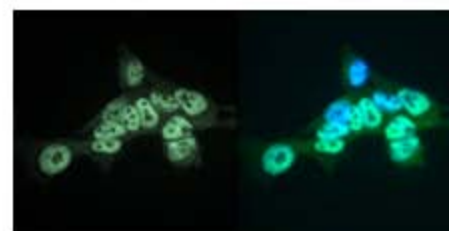

60 MINUTES

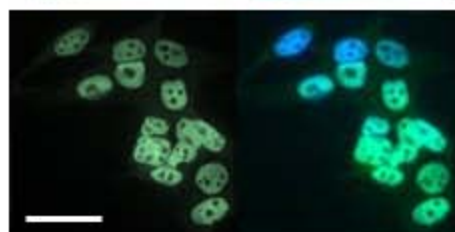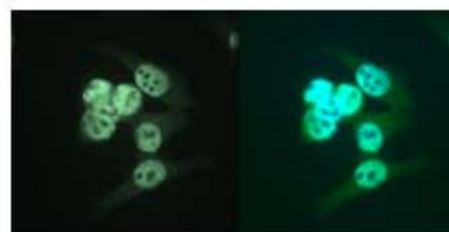

Supplement: Supplementary Data [file supp_JOE-14-0456_Supplementary_figure_1.pdf]

10% SERUM

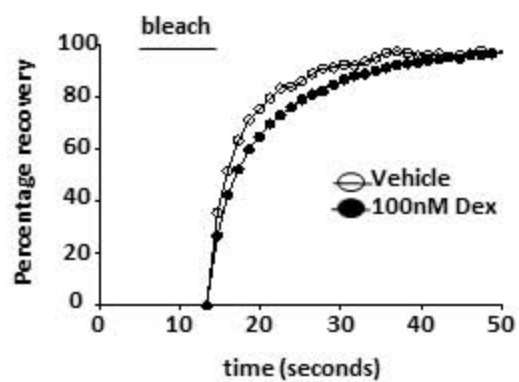

50% SERUM

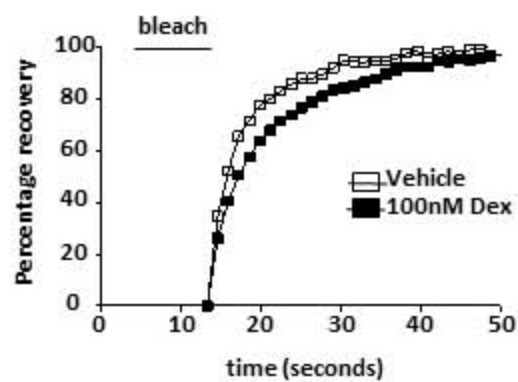

Supplement: Supplementary Data [file supp_JOE-14-0456_Supplementary_figure_2.pdf]

**A**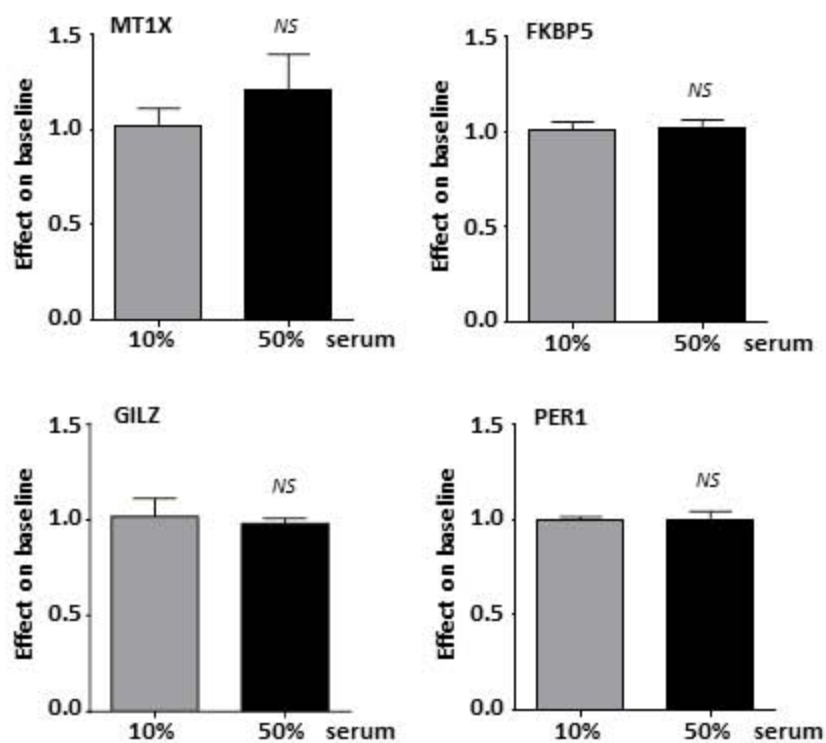**B**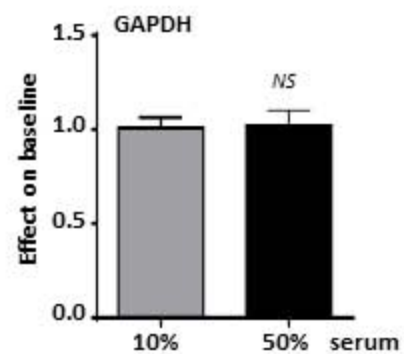**C**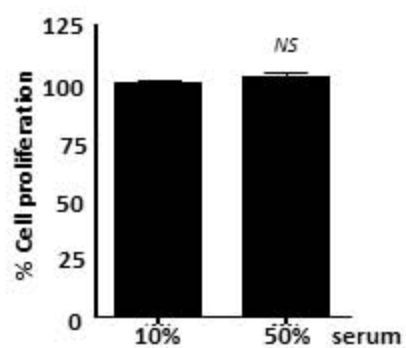

Supplement: Supplementary Data [file supp_JOE-14-0456_Supplementary_figure_3.pdf]

**A**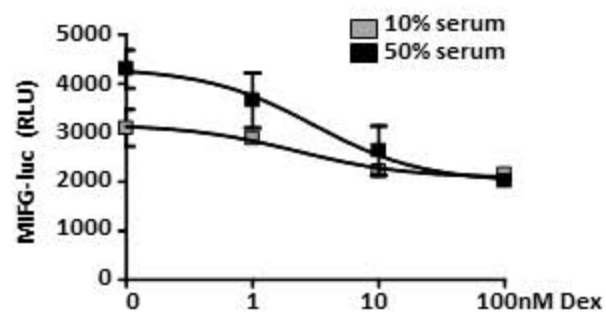**B**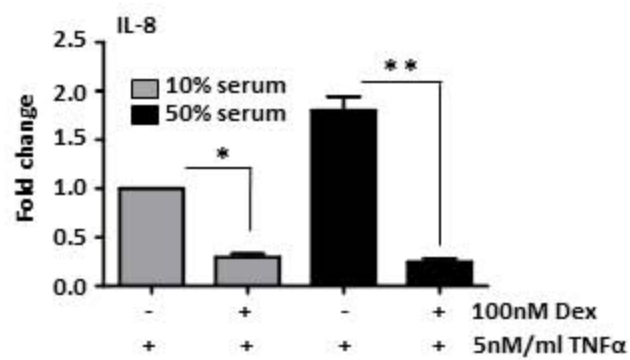

Supplement: Supplementary Data [file supp_JOE-14-0456_Supplementary_figure_4.pdf]

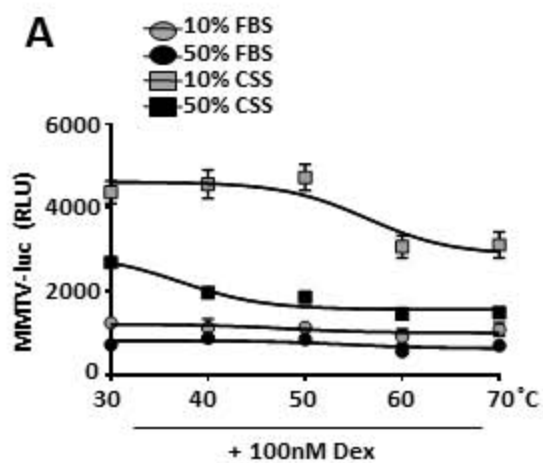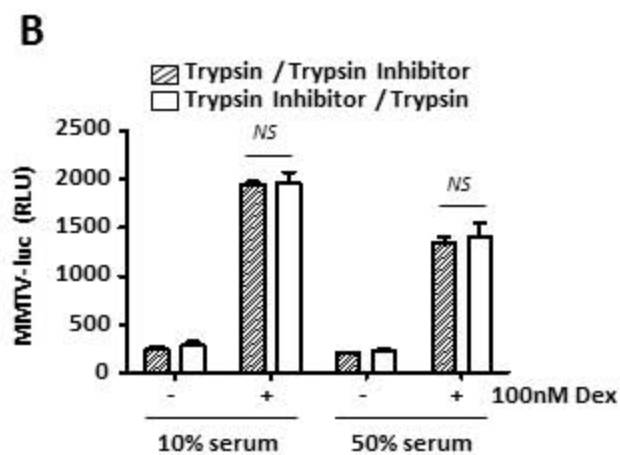

Supplement: Supplementary Data [file supp_JOE-14-0456_Supplementary_figure_5.pdf]

**A**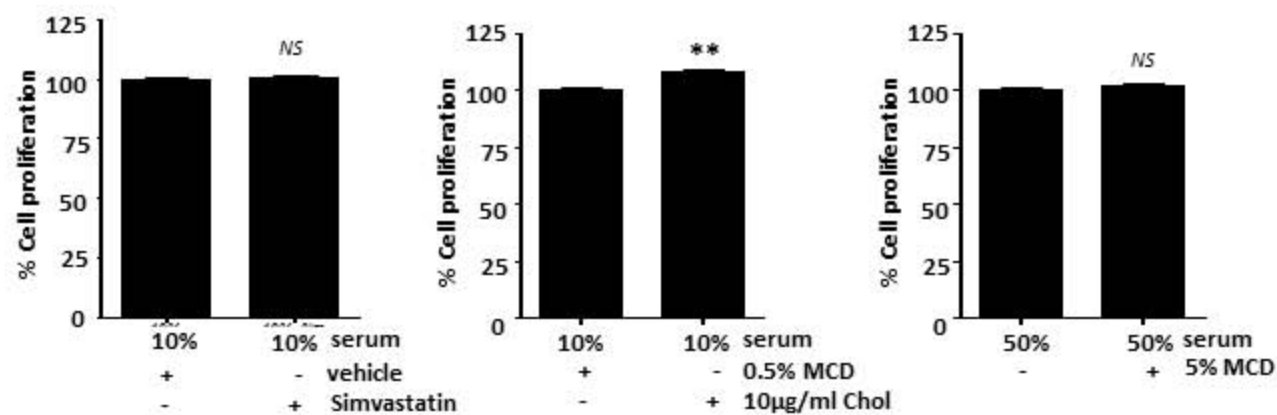**B**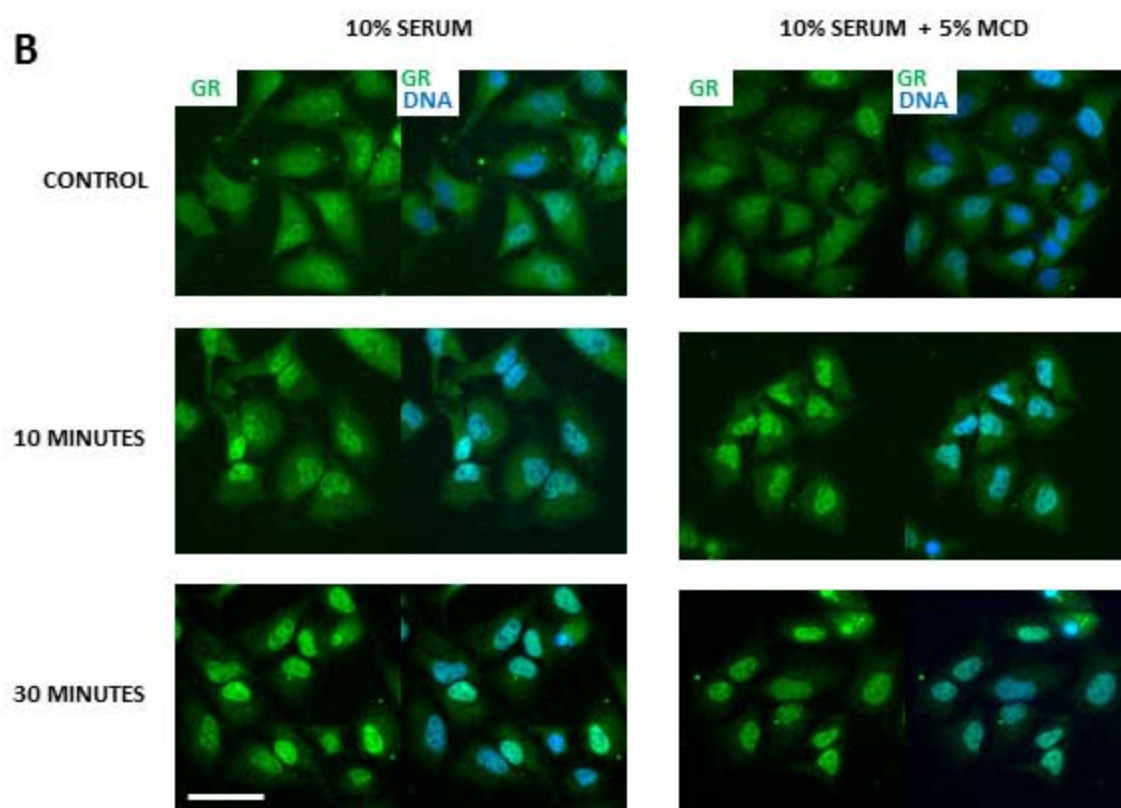

Supplement: Supplementary Data [file supp_JOE-14-0456_Supplementary_figure_6.pdf]

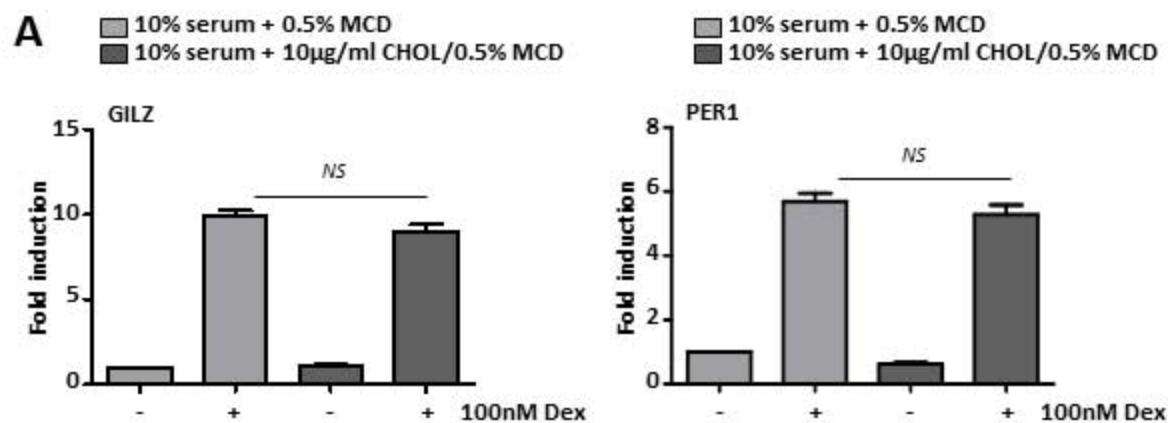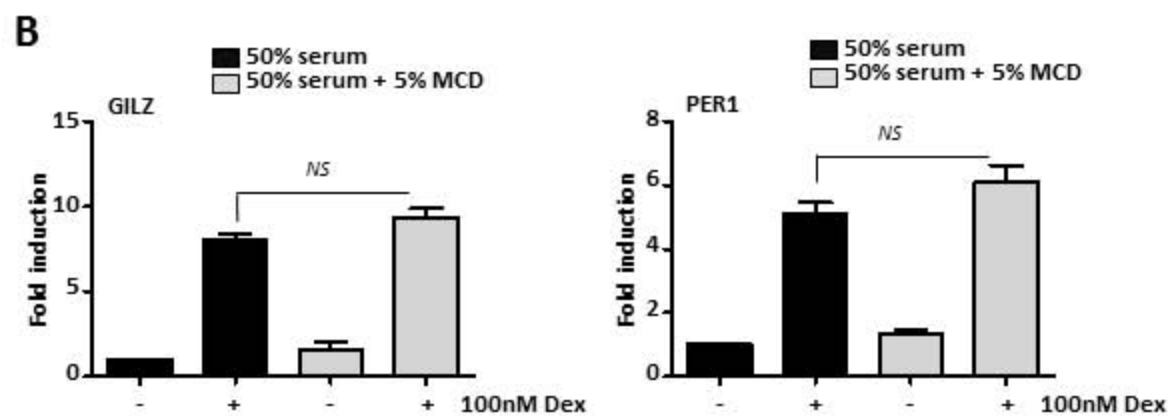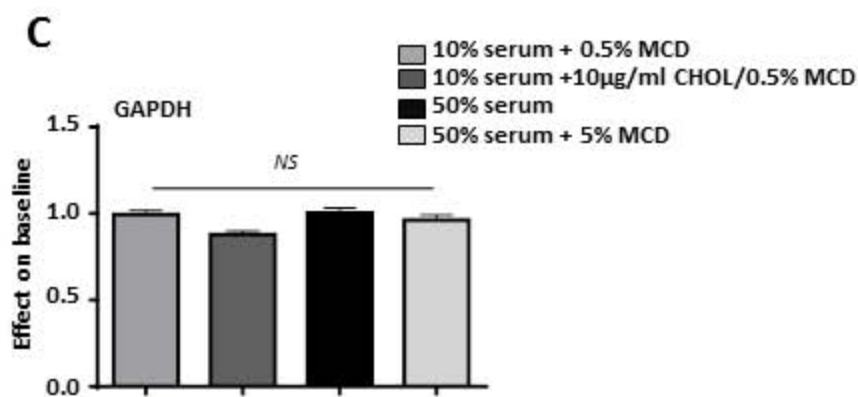

Supplement: Supplementary Data [file supp_JOE-14-0456_Supplementary_figure_7.pdf]
