# Supplementary material for: Serum cholesterol selectively regulates glucocorticoid sensitivity through activation of JNK
Source: J Endocrinol. 2014 Aug 26;223(2):155–66. doi: 10.1530/JOE-14-0456 (PMC4191185; doi:10.1530/JOE-14-0456)
Supplement: Supplementary Data [file supp_JOE-14-0456_Supplementary_table_1.pdf]

Table S1 Primer sequences used for human cells

Gene name		Primer pairs
GAPDH:	Forward	5’-GCCAGCCGAGCCACATC-3’
	Reverse	5’-GTGACCAGGCGCCCAAT-3’
β-ACTIN:	Forward	5’- ATTGGCAATGAGCGGTCCCG-3’
	Reverse	5’- AGGGCAGTGATCTCCTTCTG-3’
FKBP5:	Forward	5’-TGTCTCCCACGTGTGTATTATG-3’
	Reverse	5’-TTTGCTCAGAACCACTCACAC-3’
GILZ:	Forward	5’-TGTGGATGAGGGATGAACAA-3’
	Reverse	5’-ACCCGCTACAGACAAGCTTT-3’
MT1X:	Forward	5’-CAGCTGTGCTCTCAGATGTAAA-3’
	Reverse	5’-TGTAGCAAACGGGTCAGG-3’
PER1:	Forward	5’-CTGCACCAGCTAGACTCCATT-3’
	Reverse	5’-GGAGAAGAAAGCCTCTCATGG-3’
