# Supplementary material for: Serum cholesterol selectively regulates glucocorticoid sensitivity through activation of JNK
Source: J Endocrinol. 2014 Aug 26;223(2):155–66. doi: 10.1530/JOE-14-0456 (PMC4191185; doi:10.1530/JOE-14-0456)
Supplement: Supplementary Data [file supp_JOE-14-0456_Supplementary_table_2.pdf]

Table S2 Primer sequences used for mouse tissues

Gene name		Primer pairs
GAPDH:	Forward	5'-TGGCCTCCAAGGAGT-3'
	Reverse	5'-GGGATAGGGCCTCTC-3'
DUSP1:	Forward	5'-GGATATGAAGCGTTT-3'
	Reverse	5'-GGATTCTGCACTGTC-3'
FKBP5:	Forward	5'-CGGAAAGGCGAGGGATACTC-3'
	Reverse	5'-CGTGTACTTGCCTCCCTTGA-3'
GILZ:	Forward	5'-GGTGGCCCTAGACAA-3'
	Reverse	5'-TCTTCTCAAGCAGCT-3'
NFKBIA:	Forward	5'-GCACTTGGCAATCATCCACG-3'
	Reverse	5'-CACGTGTGGCCATTGTAGTT-3'
MT1:	Forward	5'-AGATCTCGGAATGGACCCCA-3'
	Reverse	5'-AGGAGCAGCAGCTCTTCTTG-3'
PER1:	Forward	5'-CGGCCAGGTGTCGTGATTA-3'
	Reverse	5'-CTCTGGTGGCAGTCGAAGTT-3'
